# Resilient Event-Based Fuzzy Fault Detection for DC Microgrids in Finite-Frequency Domain against DoS Attacks

**DOI:** 10.3390/s24092677

**Published:** 2024-04-23

**Authors:** Bowen Ma, Qing Lu, Zhou Gu

**Affiliations:** College of Mechanical and Electronic Engineering, Nanjing Forestry University, Nanjing 210037, China; njwyse@163.com (B.M.); qinglu@njfu.edu.cn (Q.L.)

**Keywords:** DC microgrids, T-S fuzzy model, fault detection, DoS attacks, integrated event-triggered mechanism

## Abstract

This paper addresses the problem of fault detection in DC microgrids in the presence of denial-of-service (DoS) attacks. To deal with the nonlinear term in DC microgrids, a Takagi-Sugeno (T-S) model is employed. In contrast to the conventional approach of utilizing current sampling data in the traditional event-triggered mechanism (ETM), a novel integrated ETM employs historical information from measured data. This innovative strategy mitigates the generation of additional triggering packets resulting from random perturbations, thus reducing redundant transmission data. Under the assumption of faults occurring within a finite-frequency domain, a resilient event-based $H_{-}/H_{\infty }$ fault detection filter (FDF) is designed to withstand DoS attacks. The exponential stability conditions are derived in the form of linear matrix inequalities to ensure the performance of fault detected systems. Finally, the simulation results are presented, demonstrating that the designed FDF effectively detects finite-frequency faults in time even under DoS attacks. Furthermore, the FDF exhibits superior fault detection sensitivity compared to the conventional $H_{\infty }$ method, thus confirming the efficacy of the proposed approach. Additionally, it is observed that a trade-off exists between fault detection performance and the data releasing rate (DRR).

## 1. Introduction

With the development of renewable energy, DC microgrids have attracted increasing attention due to the advantage of enabling customers to maintain electrical service independently of the main grid [1]. Renewable energy sources are effectively utilized in a DC microgrid through some structural devices, such as solar panels, wind turbines, and batteries for energy storage. Such microgrids, therefore, can offer flexibility to operate autonomously or in tandem with the main electrical grid, facilitating localized power generation and distribution. Compared with traditional AC microgrids, DC microgrids have many distinct merits, such as enhanced efficiency, seamless integration, and improved compatibility with consumer electronics [2,3].

It is well known that constant power loads (CPLs) play a significant role in DC microgrids because of the adverse effects on the nonlinearity degree and stability of the overall system [4,5]. The nonlinear dynamics and negative incremental impedance of CPLs may contribute to the deterioration of system performance and even system paralysis. To address this, scholars have explored various methods, among which the T-S fuzzy method can effectively approximate nonlinearities by a convex sum of local linear systems [6,7], facilitating quantities of interesting research on CPLs of DC microgrids [8,9]. Regional nonlinear methods are also used for system stability analysis [10,11]. In addition, as the environment changes or the fault occurs, the system sometimes enters different modes as in [12]; therefore, a fuzzy switching model is required to describe this behavior [13,14].

Owing to the low impedance characteristic of DC microgrid systems, the capacitive filters linked with the converters swiftly discharge during a fault occurrence, leading to significant current surges within a short duration. If the fault cannot be solved promptly and is not isolated from the microgrid systems, it may contribute to system instability or even damage to the converter. Consequently, a fault detection filter (FDF) is designed to capture changes in the system behavior by using measured outputs [15,16]. Note that faults often occur within specific limited frequency bands in practical applications [17], and to the best of the authors’ knowledge, little attention has been paid to the fault detection problem for DC microgrids in the finite-frequency domain, which motivates this research.

The dispersed components in DC microgrids typically interact via a communication network [18]. Even though the introduction of a communication network brings low cost and high reliability, network resources are limited. Therefore, developing a suitable data communication scheme is necessary to improve resource utilization without compromising the system performance of DC microgrids. The event-triggered mechanism (ETM) has been widely employed in the study of DC microgrids. Under an ETM, only the measurements meeting a certain triggering condition can be released, thus reducing the amount of transmission data. Applying such a communication scheme, the distributed secondary control problem of DC microgrids with a single bus was solved in [19]. In [20], a distributed self-triggered algorithm was developed for islanded microgrids, which greatly reduced the computation costs. A memory-based ETM that included historical discrete sampling data was investigated for intelligent vehicle transportation systems in [21]. Event-triggered data-driven control was proposed for unknown interconnected systems in [22].

The openness and sharing of communication networks results in potential challenges, such as data packet loss, network-introduced time delay, and malicious cyber-attacks. Cyber attacks, such as deception attacks and DoS attacks, can compromise the electric quality of microgrid systems or even lead to system collapse. DoS attackers launch an attack by occupying the bandwidth of the communication network, thus interrupting signal transmission. In this case, it leads to system stability or even paralysis. Consequently, a large amount of research has been conducted on mitigating the impacts of DoS attacks. For instance, event-based control strategies were developed for DC microgrids in the presence of intermittent DoS attacks, as demonstrated in [23]. Studies in [24] explored the consensus control of multi-agent systems against DoS attacks with specific constraints on frequency and duration. The authors in [25] further investigated control systems under DoS attacks, specifically focusing on scenarios where the end of DoS attacks does not coincide with sampling times. Resilient control strategies were developed for nonlinear multi-agent systems to mitigate the affect of DoS attacks in [26].

This paper focuses on finite-frequency fault detection for event-triggered DC microgrids using T-S fuzzy rules, considering DoS attacks. The main contributions of this work are outlined below:

(1) A novel integrated ETM is proposed for DC microgrids, under which historical state information is utilized to design the triggering condition. This ETM generates fewer events compared to the traditional event-triggered mechanism (traditional ETM), while ensuring the performance of fault detection for DC microgrids.
(2) An integrated event-triggered fault detection filter (FDF) is designed for DC microgrids under DoS attacks. In contrast to existing fault detection methods for DC microgrids, the frequency band of fault occurrence is considered in the proposed FDF, which reduces the constructiveness of the filter design.

The remainder of this paper is organized as follows: Section 2 gives the problem formulation. Section 3 presents the $H_{-}/H_{\infty }$ FDF design method, along with sufficient conditions. In Section 4, convincing simulation results are presented. Our work is concluded in Section 5.

## 2. Problem Formulation

### 2.1. System Modeling

**Figure 1:**
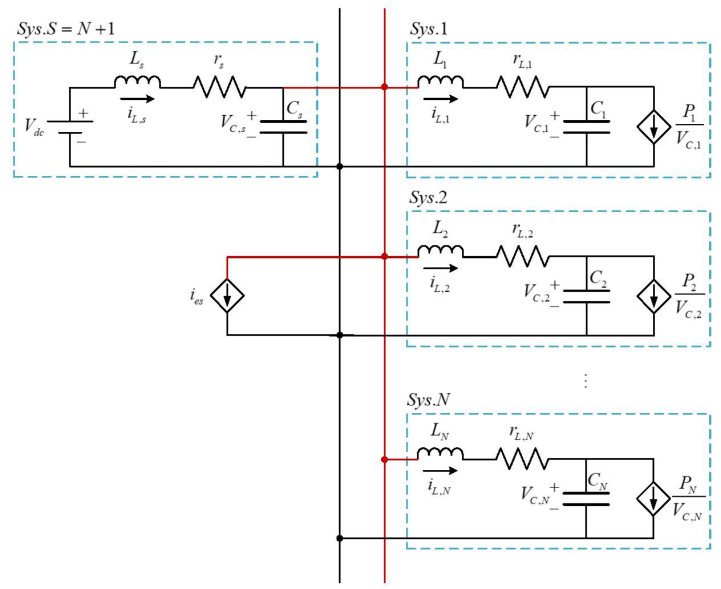
Circuit diagram of DC microgrids.

Figure 1 shows the typical circuit diagram of a DC microgrid, which consists of multiple subsystems with *N* CPLs and one energy storage system (ESS) connected with the direct current source $V_{dc}$. For convenience, all the relevant physical variables in this paper are denoted similarly to [23,27]. Define $x_{k}\left(t\right)={\left[i_{L,k}\;v_{C,k}\right]}^{T}$, where $i_{L,k}$ denotes the current of the inductor, and $v_{C,k}$ is the voltage of the capacitor. Then, one can obtain the following state function of the *k*th CPL:

(1) $$\begin{array}{c} {\dot{x}}_{k}\left(t\right)=A_{k}x_{k}\left(t\right)+g_{k}\nu_{k}\left(x_{k}\left(t\right)\right)+A_{ks}x_{s}\left(t\right),\;k\in \left\{1,2,\cdots ,N\right\}, \end{array}$$

where

$$A_{k}=\left[\begin{array}{cc} -\frac{r_{L,k}}{L_{k}} & -\frac{1}{L_{k}}\\ \frac{1}{C_{k}} & 0 \end{array}\right],\;g_{k}=\left[\begin{array}{c} 0\\ -\frac{P_{k}}{C_{k}} \end{array}\right],\;A_{ks}=\left[\begin{array}{cc} 0 & \frac{1}{L_{k}}\\ 0 & 0 \end{array}\right],\;\nu_{k}\left(x_{k}\left(t\right)\right)=\frac{1}{v_{C,k}}.$$

Assume that all the CPLs are ideal, i.e., the power $P_{k}$ remains unchanged, and the DC microgrid has been stabilized by the energy storage current $i_{es}\left(t\right)$. Then, we can obtain

(2) $${\dot{x}}_{s}\left(t\right)=A_{s}x_{s}\left(t\right)+b_{s}V_{dc}+b_{es}i_{es}\left(t\right)+\sum_{k=1}^{N}\iota x_{k}\left(t\right),$$

where

$$A_{s}=\left[\begin{array}{cc} -\frac{r_{s}}{L_{s}} & -\frac{1}{L_{s}}\\ \frac{1}{C_{s}} & 0 \end{array}\right],\;\iota =\left[\begin{array}{cc} 0 & 0\\ -\frac{1}{C_{s}} & 0 \end{array}\right],\;b_{s}=\left[\begin{array}{c} \frac{1}{L_{s}}\\ 0 \end{array}\right],\;b_{es}=\left[\begin{array}{c} 0\\ -\frac{1}{C_{s}} \end{array}\right].$$

Using the method of shifting the equilibrium point as in [5], the DC microgrid with *N* CPLs and one ESS can be formulated as

(3) $$\begin{array}{c} \dot{\tilde{x}}\left(t\right)=A\tilde{x}\left(t\right)+DH\left(\tilde{x}\left(t\right)\right)+B_{es}{\tilde{i}}_{es}\left(t\right), \end{array}$$

where ${\tilde{i}}_{es}\left(t\right)$ is designed by ${\tilde{i}}_{es}=\tilde{K}\tilde{x}\left(t\right)$ with a preset control gain $\tilde{K}$, and

$$\begin{array}{cc} \tilde{x}\left(t\right) & =\mathrm{col}\left\{{\tilde{x}}_{1}\left(t\right),{\tilde{x}}_{2}\left(t\right)\cdots {\tilde{x}}_{N}\left(t\right),{\tilde{x}}_{s}\left(t\right)\right\}=x\left(t\right)-x_{e},\\ H(\tilde{x}) & =\mathrm{col}\left\{\nu_{1}\left({\tilde{x}}_{1}\left(t\right)\right),\nu_{2}\left({\tilde{x}}_{2}\left(t\right)\right),\cdots ,\nu_{N}\left({\tilde{x}}_{N}\left(t\right)\right)\right\},\;B_{es}=\mathrm{col}\left\{0,\cdots ,0,b_{es}\right\},\\ A & =\left[\begin{array}{ccccc} A_{1} & 0 & \cdots & 0 & A_{1s}\\ 0 & A_{2} & \cdots & 0 & A_{2s}\\ \vdots & \vdots & \ddots & \vdots & \vdots \\ 0 & 0 & \cdots & A_{N} & A_{Ns}\\ \iota & \iota & \cdots & \iota & A_{s} \end{array}\right],\;D=\left[\begin{array}{cccc} g_{1} & 0 & \cdots & 0\\ 0 & g_{2} & \cdots & 0\\ \vdots & \vdots & \ddots & \vdots \\ 0 & 0 & \cdots & g_{N}\\ 0 & 0 & \cdots & 0 \end{array}\right], \end{array}$$

wherein $\nu_{k}\left({\tilde{x}}_{k}\left(t\right)\right)={\tilde{v}}_{C,k}/v_{c0,k}\left({\tilde{v}}_{C,k}+v_{c0,k}\right)$; $x_{e}$ and $v_{c0,k}$ denote the equilibrium point of DC microgrids and $v_{c,k}$, respectively.

As demonstrated in [28], the multiple CPLs in a DC microgrid can be transformed into only one equivalent CPL. Therefore, only one CPL is considered in this study.

Inspired by [27], assume the nonlinear term $\nu_{1}/{\tilde{v}}_{C,1}$ is bounded by $V_{min}\leq \nu_{1}/{\tilde{v}}_{C,1}\leq V_{max}$ for a given local region $R_{1,\tilde{x}}=\left\{\tilde{x}|-w_{1,1}\leq {\tilde{i}}_{L,1}\leq w_{1,1},-w_{2,1}\leq {\tilde{v}}_{c,1}\leq w_{2,1}\right\}$, where

(4) $$\begin{array}{c} V_{min}=\frac{1}{v_{c0,1}\left({\tilde{w}}_{2,1}+v_{c0,1}\right)},\;V_{max}=\frac{1}{v_{c0,1}\left(-{\tilde{w}}_{2,1}+v_{c0,1}\right)}. \end{array}$$

Applying the sector nonlinearity approach, $\nu_{1}/{\tilde{v}}_{C,1}$ is expressed by

(5) $$\begin{array}{c} \left\{\begin{array}{c} \frac{\nu_{1}}{{\tilde{v}}_{C,1}}=M_{1}V_{\min}+M_{2}V_{\max}\\ M_{1}+M_{2}=1 \end{array}\right., \end{array}$$

where $M_{1}$ and $M_{2}$ are normalized membership functions.

Choose $\nu_{1}/{\tilde{v}}_{C,1}$ as the premise variable θ(t), and define the membership functions as $\varphi_{i}\left(\theta \left(t\right)\right)\left(i\in \left\{1,2\right\}\right)$. By solving (5), we have

(6) $$\begin{array}{c} \left\{\begin{array}{c} \varphi_{1}\left(\theta \left(t\right)\right)=\frac{V_{\max}{\tilde{v}}_{C,1}-\nu_{1}}{\left(V_{\max}-V_{\min}\right){\tilde{v}}_{C,1}}\\ \varphi_{2}\left(\theta \left(t\right)\right)=\frac{\nu_{1}-V_{\min}{\tilde{v}}_{C,1}}{\left(V_{\max}-V_{\min}\right){\tilde{v}}_{C,1}} \end{array}\right.. \end{array}$$

Taking the external disturbance $\rho \left(t\right)\in L_{2}\left[0,+\infty \right)$ and actuator fault f(t) into account, the ith fuzzy rule is given by:

Rule *i*: If $\theta_{1}\left(t\right)$ is $F_{i1}$, ⋯, $\theta_{r}\left(t\right)$ is $F_{ir}$, then

(7) $$\begin{array}{c} \left\{\begin{array}{c} \dot{\tilde{x}}\left(t\right)={\bar{A}}_{i}\tilde{x}\left(t\right)+B_{1i}\rho \left(t\right)+B_{2i}f\left(t\right)\\ y\left(t\right)=C_{i}\tilde{x}\left(t\right) \end{array}\right., \end{array}$$

where ${\bar{A}}_{i}={\bar{A}}_{0i}+B_{es}{\tilde{K}}_{i}$ which is Hurwitz; y(t) denotes the measurable system output; ${\tilde{K}}_{i},B_{1i},B_{2i}$, and $C_{i}$ are all known real constant matrices with appropriate dimensions and

$$\begin{array}{cc} {\bar{A}}_{01} & =\left[\begin{array}{cccc} -\frac{r_{L,1}}{L_{1}} & -\frac{1}{L_{1}} & 0 & \frac{1}{L_{1}}\\ \frac{1}{C_{1}} & \frac{P_{1}}{C_{1}}V_{\min} & 0 & 0\\ 0 & 0 & -\frac{r_{s}}{L_{s}} & -\frac{1}{L_{s}}\\ -\frac{1}{C_{s}} & 0 & \frac{1}{C_{s}} & 0 \end{array}\right],\;{\bar{A}}_{02}=\left[\begin{array}{cccc} -\frac{r_{L,1}}{L_{1}} & -\frac{1}{L_{1}} & 0 & \frac{1}{L_{1}}\\ \frac{1}{C_{1}} & \frac{P_{1}}{C_{1}}V_{\max} & 0 & 0\\ 0 & 0 & -\frac{r_{s}}{L_{s}} & -\frac{1}{L_{s}}\\ -\frac{1}{C_{s}} & 0 & \frac{1}{C_{s}} & 0 \end{array}\right]. \end{array}$$

Applying the same technique in [25], the T-S fuzzy system can be expressed by:

(8) $$\begin{array}{c} \left\{\begin{array}{c} \dot{\tilde{x}}\left(t\right)=\sum_{i=1}^{2}\varphi_{i}\left(\theta \left(t\right)\right)\left[{\bar{A}}_{i}\tilde{x}\left(t\right)+B_{1i}\rho \left(t\right)+B_{2i}f\left(t\right)\right]\\ y\left(t\right)=\sum_{i=1}^{2}\varphi_{i}\left(\theta \left(t\right)\right)C_{i}\tilde{x}\left(t\right) \end{array}\right.. \end{array}$$

Moreover, according to [29], the finite-frequency fault f(t) can be described as:

(9) $$\begin{array}{c} \varpi :=\left\{\omega \in |\tau \left(\omega -\omega_{1}\right)\left(\omega -\omega_{2}\right)\leq 0\right\}, \end{array}$$

where ω is the frequency of f(t), which can be categorized into the following cases:

(1) τ = 1, $-\omega_{1}=\omega_{2}$, f(t) occurs in the low-frequency band.
(2) τ = 1, $0\leq \omega_{2}-\omega_{1}<2\pi$, f(t) occurs in the middle-frequency band.
(3) τ = −1, $-\omega_{1}=\omega_{2}$, f(t) occurs in the high-frequency band.

**Remark** **1.** *Unlike general fault detection approaches of DC microgrids, the fault occurrence in the finite-frequency domain is considered in this paper, reducing conservativeness in the filter design.*

Before designing the fuzzy FDF for DC microgrids, a new premise variable $\hat{\theta }\left(t\right)$ is needed since the premise variable between the system and the FDF is actually asynchronous, which is assumed to satisfy ${\hat{\varphi }}_{j}-k_{j}\varphi_{j}\geq 0\left(0<\kappa_{j}\leq 1\right)$ [30]. For brevity, $\varphi_{i}\left(\theta \left(t\right)\right)$ and $\varphi_{j}\left(\hat{\theta }\left(t\right)\right)$ are denoted by $\varphi_{i}$ and ${\hat{\varphi }}_{j}$, respectively. Then, similarly to (8), the FDF is represented by

(10) $$\begin{array}{c} \left\{\begin{array}{c} \dot{\hat{x}}\left(t\right)=\sum_{j=1}^{2}{\hat{\varphi }}_{j}\left[A_{fj}\hat{x}\left(t\right)+B_{fj}y_{f}\left(t\right)\right]\\ r\left(t\right)=\sum_{j=1}^{2}{\hat{\varphi }}_{j}\left[C_{fj}\hat{x}\left(t\right)+D_{fj}y_{f}\left(t\right)\right] \end{array}\right., \end{array}$$

where $\hat{x}\left(t\right)$ is the filter state vector; $y_{f}\left(t\right)$ is the filter input; r(t) is the generated residual signal; $A_{fj}$, $B_{fj}$, $C_{fj}$ and $D_{fj}$ are the filter gain matrices with proper dimensions to be designed.

### 2.2. DoS Attacks and the ETM Design

In this study, a general model of DoS attacks with a fixed period *T* is considered [31]:

(11) $$\begin{array}{c} S_{dos}=\left\{\begin{array}{cc} 1, & t\in [nT,nT+T_{\mathrm{off}}\left(n\right))\\ 0, & t\in [nT+T_{\mathrm{off}}\left(n\right),nT+T), \end{array}\right. \end{array}$$

where $T_{\mathrm{off}}\left(n\right)$ is lower-bounded by $T_{\mathrm{off}}^{\min}$ due to power constraints. The whole attack period includes a sleeping period $\left[nT,nT+T_{\mathrm{off}}\left(n\right)\right)$ and an active period $\left[nT+T_{\mathrm{off}}\left(n\right),nT+T\right)$. By defining $k_{n}=\sup\left\{k\in N|nT+T_{\mathrm{off}}\left(n\right)\geq t_{k,n}\right\}$, $l_{n}=\sup\left\{l\in N|nT+T_{\mathrm{off}}\left(n\right)\geq t_{k,n}+lh\right\}$, and considering the historical information, the integrated ETM can be obtained:

(12) $$\begin{array}{cc} \; & {\left[{\bar{y}}_{m}\left(t_{k,n}+lh\right)-{\bar{y}}_{m}\left(t_{k,n}\right)\right]}^{T}\Omega \left[{\bar{y}}_{m}\left(t_{k,n}+lh\right)-{\bar{y}}_{m}\left(t_{k,n}\right)\right]\\ \; & >\varepsilon {\bar{y}}_{m}^{T}\left(t_{k,n}+lh\right)\Omega {\bar{y}}_{m}\left(t_{k,n}+lh\right),\;k\in \left\{0,1,\cdots ,k_{n}\right\},\;l\in \left\{1,2,\cdots ,l_{n}\right\}, \end{array}$$

where $t_{k,n}$ denotes the real transmitted instant, and ${\bar{y}}_{m}\left(t_{k,n}\right)$ denotes the output of the event generator averaged by the integral, which is defined as

(13) $$\begin{array}{c} {\bar{y}}_{m}\left(t_{k,n}\right)=\frac{1}{T}\int_{t_{k,n}-T}^{t_{k,n}}y\left(s\right)ds. \end{array}$$

Adopting Simpson’s rule in [32], one has

(14) $$\begin{array}{c} \frac{1}{T}\int_{t_{k,n}-T}^{t_{k,n}}y\left(s\right)ds\approx \frac{1}{6}y\left(t_{k,n}\right)+\frac{2}{3}y\left(t_{k,n}-\frac{T}{2}\right)+\frac{1}{6}y\left(t_{k,n}-T\right), \end{array}$$

where T is a preset integral period.

Combining (12) and (13) yields the actual input of the FDF with the proposed integrated ETM:

(15) $$\begin{array}{c} y_{f}\left(t\right)=\left\{\begin{array}{cc} {\bar{y}}_{m}\left(t_{k,n}\right), & t\in H_{k,n}\cap L_{1,n}\\ 0, & t\in L_{2,n}, \end{array}\right. \end{array}$$

where $H_{k,n}=\left[t_{k,n},t_{k+1,n}\right)$; $L_{1,n}$ and $L_{2,n}$ stand for $\left[nT,nT+T_{\mathrm{off}}\left(n\right)\right)$ and $\left[nT+T_{\mathrm{off}}\left(n\right),nT+T\right)$, respectively.

**Remark** **2.** *In (13), when the integral period T sets to be zero, the proposed integrated ETM becomes a normal traditional ETM, as in [33,34].*

### 2.3. T-S Fuzzy Switched Residual System

For technical convenience, define the following continuous intervals:

$$\begin{array}{c} \left\{\begin{array}{c} F_{n}^{0}=\left[t_{k,n},t_{k,n}+h\right),\\ F_{n}^{l}=\left[t_{k,n}+lh,t_{k,n}+lh+h\right),\\ F_{n}^{l_{n}}=\left[t_{k,n}+l_{n}h,nT+T_{\mathrm{off}}\left(n\right)\right). \end{array}\right. \end{array}$$

Then, for $t\in L_{1,n}$, the following definition is presented:

$$\begin{array}{c} \left\{\begin{array}{c} \eta_{k,n}\left(t\right)=\left\{\begin{array}{cc} t-t_{k,n}, & t\in F_{n}^{0}\\ t-t_{k,n}-lh, & t\in F_{n}^{l}\\ t-t_{k,n}-l_{n}h, & t\in F_{n}^{l_{n}} \end{array}\right.\\ e_{k,n}=\left\{\begin{array}{cc} 0, & t\in F_{n}^{0}\\ y\left(t_{k,n}\right)-y\left(t_{k,n}+lh\right), & t\in F_{n}^{l}\\ y\left(t_{k,n}\right)-y\left(t_{k,n}+l_{n}h\right), & t\in F_{n}^{l_{n}}. \end{array}\right. \end{array}\right. \end{array}$$

For $t\in L_{2,n}$, DoS attacks are active; in this case, $\eta_{k,n}\left(t\right)=e_{k,n}=0$.

Define $\eta_{k,n}\left(t\right)=\epsilon$, and $\tau_{g,n}=\left(g-1\right)T_{\mathrm{off}}\left(n\right)+nT$(g=1,2) for brevity. Based on the above discussion, denote $\psi \left(t\right)=\mathrm{col}\{\tilde{x}\left(t\right),\hat{x}\left(t\right)\}$, ϑ(t)=col{ρ(t),f(t)}, and $r_{e}\left(t\right)=r\left(t\right)-f\left(t\right)$; then, one can derive the following switched augmented system: for $t\in [\tau_{g,n},\tau_{3-g,n+g-1})$,

(16) $$\begin{array}{c} \left\{\begin{array}{c} \dot{\psi }\left(t\right)=\sum_{i=1}^{2}\sum_{i=1}^{2}\varphi_{i}{\hat{\varphi }}_{j}[A_{1,ij}\psi \left(t\right)+A_{2,ij}^{g}\psi \left(t-\epsilon \right)+\beta \vartheta \left(t\right)+E_{1,j}^{g}e_{k,n}\left(t\right)\\ \;\;\;\;\;+A_{3,ij}^{g}\psi \left(t-\epsilon -\frac{T}{2}\right)+A_{4,ij}^{g}\psi \left(t-\epsilon -T\right)]\\ r_{e}\left(t\right)=\sum_{i=1}^{2}\sum_{i=1}^{2}\varphi_{i}{\hat{\varphi }}_{j}[C_{1,j}\psi \left(t\right)+C_{2,ij}^{g}\psi \left(t-\epsilon \right)+\bar{H}\vartheta \left(t\right)+E_{2,j}^{g}e_{k,n}\left(t\right)\\ \;\;\;\;\;+C_{3,ij}^{g}\psi \left(t-\epsilon -\frac{T}{2}\right)+C_{4,ij}^{g}\psi \left(t-\epsilon -T\right)] \end{array}\right., \end{array}$$

where

$$\begin{array}{cc} A_{1,ij} & =\left[\begin{array}{cc} {\bar{A}}_{i} & 0\\ 0 & A_{fj} \end{array}\right],\;A_{d+1,ij}^{1}=\left[\begin{array}{cc} 0 & 0\\ n_{d}B_{fj}C_{i} & 0 \end{array}\right]\left(d=1,2,3\right),\;\bar{B}=\left[B_{1}\;B_{2}\right],\\ \beta_{i} & =\left[\begin{array}{c} {\bar{B}}_{i}\\ 0 \end{array}\right],\;E_{1,j}^{1}=\left[\begin{array}{c} 0\\ B_{fj} \end{array}\right],\;C_{1,j}=\left[0\;C_{f}\right],\;C_{l+1,ij}^{1}=\left[n_{l}D_{fj}C_{i}\;0\right]\\ E_{2,j}^{1} & =D_{fj},\;\bar{H}=\left[\begin{array}{cc} 0 & -I \end{array}\right],\;A_{l+1,ij}^{2}=C_{l+1,ij}^{2}=E_{1,2}=E_{2,2}=0\\ n_{1} & =\frac{1}{6},\;n_{2}=\frac{2}{3},\;n_{3}=\frac{1}{6}. \end{array}$$

In order to detect the occurrence of actuator faults, the following evaluation function χ(t) is constructed:

(17) $$\begin{array}{c} \chi \left(t\right)=\sqrt{\int_{0}^{t}r^{T}\left(s\right)r\left(s\right)ds}, \end{array}$$

with the threshold $\chi_{th}$ chosen as

(18) $$\begin{array}{c} \chi_{th}=\underset{\rho \left(t\right)\in L_{2},f\left(t\right)=0}{\sup}\chi \left(t\right), \end{array}$$

and the fault is detected by

(19) $$\begin{array}{c} \left\{\begin{array}{c} \chi \left(t\right)>\chi_{th}\Rightarrow \mathrm{faulty}\\ \chi \left(t\right)\leq \chi_{th}\Rightarrow \mathrm{fault}\;\mathrm{free}. \end{array}\right. \end{array}$$

In this article, the main purpose of this paper is to design an $H_{-}/H_{\infty }$ FDF such that

1. When ϑ(t)≡0, the system (16) achieves exponential stability.
2. Under zero initial conditions, when ρ(t)≡0,f(t)≠0, the $H_{-}$ fault sensitivity condition
  (20) $$\begin{array}{c} \int_{0}^{\infty }r_{e}^{T}\left(t\right)r_{e}\left(t\right)dt\geq \beta^{2}\int_{0}^{\infty }f^{T}\left(t\right)f\left(t\right)dt \end{array}$$
  holds for all solutions of (16) satisfying
  (21) $$\begin{array}{c} \int_{0}^{\infty }\tau \left(\omega_{1}\psi \left(t\right)+j\dot{\psi }\left(t\right)\right){\left(\omega_{2}\psi \left(t\right)+j\dot{\psi }\left(t\right)\right)}^{*}dt\leq 0, \end{array}$$
  where the asterisk * denotes the conjugate transpose.
3. Under zero initial conditions, when f(t)≡0,ρ(t)≠0, the system (16) is $H_{\infty }$ bounded by
  (22) $$\begin{array}{c} \int_{0}^{\infty }r_{e}^{T}\left(t\right)r_{e}\left(t\right)dt\leq \gamma^{2}\int_{0}^{\infty }\rho^{T}\left(t\right)\rho \left(t\right)dt. \end{array}$$

In what follows, some crucial lemmas are presented to help obtain the main results.

**Lemma** **1** ([35]). *If there exist a matrix $M=\left[\begin{array}{cc} R & G\\ {}* & R \end{array}\right]\geq 0$, R>0, scalars 0≤d(t)≤d, and a vector function $\dot{x}:\left[-d,0\right]\to R^{n}$, the following inequality*

(23) $$\begin{array}{c} -d\int_{t-d}^{t}{\dot{x}}^{T}\left(s\right)R\dot{x}\left(s\right)ds\leq O^{T}\left(t\right)ZO\left(t\right) \end{array}$$

*holds with*

$$\begin{array}{cc} O^{T}\left(t\right) & =[x^{T}\left(t\right)\;x^{T}\left(t-d\left(t\right)\right)\;x^{T}\left(t-d\right)]\\ Z & =\left[\begin{array}{ccc} -R & R-G & G\\ {}* & {\left[G-R\right]}_{s} & R-G\\ {}* & * & -R \end{array}\right]. \end{array}$$

**Lemma** **2** ([36]). *Suppose f(t)∈R is integrable, then*

(24) $$\begin{array}{c} \int_{-\infty }^{+\infty }{\left||f\left(t\right)|\right|}^{2}dt=\frac{1}{2\pi }\int_{-\infty }^{+\infty }{\left||F\left(jw\right)|\right|}^{2}dw \end{array}$$

*holds, where F(jw) is the Fourier transform of f(t).*

**Lemma** **3** ([36]). *If X is a complex Hermitian matrix, X<0 is equivalent to*

(25) Re(X)Im(X)−Im(X)Re(X)<0.

## 3. Main Results

**Theorem** **1.** *For given positive constants T,* Ω*, $T_{\mathrm{off}}^{\min}$, $h<T_{\mathrm{off}}^{\min}$, π, ε, γ, α, β, $k_{i}$(i=1,2), and $\mu_{g}>1$(g=1,2), the switched system (16) is exponentially stable with an $H_{\infty }$ attenuation level γ and an $H_{-}$ index β, if positive symmetric matrices $Q_{f}^{H}$, $P_{g}$, $Q_{gm}$, $R_{gm}$, $G_{gm}$(m=0,1,2), and matrices $M_{i}^{gr}$(r=1,2), $A_{fj}$, $B_{fj}$, $C_{fj}$, $D_{fj}$(j=1,2) with proper dimensions exist, such that the following inequalities hold:*

(26) $$\begin{array}{ll} \; & \Pi_{ij}^{gr}<0,\;k_{i}\Pi_{ij}^{gr}+k_{j}\Pi_{ji}^{gr}+M_{i}^{gr}+M_{j}^{gr}<0\;\left(i<j\right), \end{array}$$

(27) $$\begin{array}{ll} \; & \left[\begin{array}{cc} R_{gm} & G_{gm}\\ {}* & R_{gm} \end{array}\right]>0, \end{array}$$

(28) $$\begin{array}{ll} \; & \left\{\begin{array}{c} P_{g}\leq \mu_{3-g}P_{3-g}\\ Q_{gm}\leq \mu_{3-g}Q_{(3-g)m}\\ R_{gm}\leq \mu_{3-g}R_{(3-g)m} \end{array}\right.,\;\alpha T-\mathrm{In}\left(\mu_{1}\mu_{2}\right)>0, \end{array}$$

*where*

$$\begin{array}{cc} \Psi_{ij}^{gr} & =\left[\begin{array}{ccc} \Phi_{ij}^{gr} & \Delta_{ij}^{g} & \Xi_{ij}^{g^{T}}\\ {}* & -R_{g} & 0\\ {}* & * & -I \end{array}\right],\;\Pi_{ij}^{gr}=\Psi_{ij}^{gr}-M_{i}^{gr},\\ \Phi_{ij}^{1r} & =\left[\begin{array}{ccccc} \Gamma_{11ij}^{1r} & \Gamma_{12ij}^{1r} & \Gamma_{13}^{1r} & \Gamma_{14i}^{1r} & \Gamma_{15j}^{1r}\\ {}* & \Gamma_{22i}^{1r} & \Gamma_{23}^{1r} & 0 & 0\\ {}* & * & \Gamma_{33}^{1r} & 0 & 0\\ {}* & * & * & \Gamma_{44}^{1r} & 0\\ {}* & * & * & * & -\Omega \end{array}\right],\;\Phi_{ij}^{2r}=\left[\begin{array}{cccc} \Gamma_{11ij}^{2r} & \Gamma_{12ij}^{2r} & \Gamma_{13}^{2r} & \Gamma_{14i}^{2r}\\ {}* & \Gamma_{22}^{2r} & \Gamma_{23}^{2r} & 0\\ {}* & * & \Gamma_{33}^{2r} & 0\\ {}* & * & * & \Gamma_{44}^{2r} \end{array}\right],\\ \Lambda_{ij}^{1} & =\left[A_{1,ij}\;{\hat{A}}_{ij}\;0\;\beta_{1}\;E_{1,j}^{1}\right],\;\Lambda_{ij}^{2}=\left[A_{1,ij}\;0\;0\;\beta_{2}\right],\\ \Xi_{ij}^{1} & =\left[C_{1,j}\;{\hat{C}}_{ij}\;0\;\bar{H}\;E_{2,j}^{1}\right],\;\Xi_{ij}^{2}=\left[C_{1,j}\;0\;0\;\bar{H}\right],\\ \Delta_{ij}^{g} & =\left[h_{0}\Lambda_{ij}^{g^{T}}H^{T}R_{g0}\;h_{1}\Lambda_{ij}^{g^{T}}H^{T}R_{g1}\;h_{2}\Lambda_{ij}^{g^{T}}H^{T}R_{g2}\right],\;\Gamma_{11ij}^{g1}=\Gamma_{11ij}^{g2}-w_{1}w_{2}Q_{f}^{H},\\ \Gamma_{12ij}^{gr} & =\left[n_{0,ij}^{gr}\;n_{1,ij}^{gr}\;n_{2,ij}^{gr}\right],\;\Gamma_{11ij}^{g2}=\alpha P_{g}+{\left[P_{g}A_{g,ij}\right]}_{s}+\sum_{i=0}^{2}\left[Q_{gm}^{H}-e^{-\alpha h_{m}}R_{gm}^{H}\right],\\ \Gamma_{13}^{gr} & =\left[e^{-\alpha h_{0}}G_{g0}^{H}\;e^{-\alpha h_{1}}G_{g1}^{H}\;e^{-\alpha h_{2}}G_{g2}^{H}\right],\;\Gamma_{14i}^{g1}=\left[P_{g}-jw_{0}Q_{f}^{H}\right]\beta_{i},\;\Gamma_{14i}^{g2}=P_{g}\beta_{i},\\ \Gamma_{15j}^{11} & =\left[P_{1}-jw_{0}Q_{f}^{H}\right]E_{1,j}^{1},\;\Gamma_{15j}^{12}=P_{1}E_{1,j}^{1},\;\Gamma_{22}^{2r}=diag\left\{J_{0}^{2},J_{1}^{2},J_{2}^{2}\right\}\\ \Gamma_{22i}^{1r} & =\left[\begin{array}{ccc} J_{0,i}^{1} & \varepsilon n_{1}^{2}{\bar{C}}_{i}^{T}\Omega {\bar{C}}_{i} & \varepsilon n_{2}^{2}{\bar{C}}_{i}^{T}\Omega {\bar{C}}_{i}\\ {}* & J_{1,i}^{1} & \varepsilon n_{3}^{2}{\bar{C}}_{i}^{T}\Omega {\bar{C}}_{i}\\ {}* & * & J_{2i}^{1} \end{array}\right],\;\Gamma_{23}^{gr}=diag\left\{d_{g0},d_{g1},d_{g2}\right\},\\ \Gamma_{33}^{gr} & =diag\left\{r_{g0},r_{g1},r_{g2}\right\},\;\Gamma_{44}^{g1}=-\beta^{2}I,\;\Gamma_{44}^{g2}=-\gamma^{2}I,\;h_{m}=h+0.5mT,\\ n_{m,ij}^{g1} & =\left[P_{g}-jw_{0}Q_{f}^{H}\right]A_{m+2,ij}^{g}+e^{-\alpha h_{m}}\left[R_{gm}^{H}-G_{gm}^{H}\right],\;d_{gm}=e^{-\alpha h_{g}}\left[R_{gm}^{H}-G_{gm}^{H}\right]\\ n_{m,ij}^{g2} & =P_{g}A_{m+2,ij}^{g}+e^{-\alpha h_{m}}\left[R_{gm}^{H}-G_{gm}^{H}\right],\;r_{gm}=-e^{-\alpha h_{m}}\left[Q_{gm}^{H}+R_{gm}^{H}\right]\\ J_{m,i}^{1} & =e^{-\alpha h_{m}}{\left[G_{1m}^{H}-R_{1m}^{H}\right]}_{s}+n_{m+1}^{2}\varepsilon {\bar{C}}_{i}^{T}\Omega {\bar{C}}_{i},\;J_{m}^{2}=e^{-\alpha h_{m}}{\left[G_{2m}^{H}-R_{2m}^{H}\right]}_{s},\\ Q_{f}^{H} & =H^{T}Q_{f}H,\;R_{gm}^{H}=H^{T}R_{gm}H,\;Q_{gm}^{H}=H^{T}Q_{gm}H,\;G_{gm}^{H}=H^{T}G_{gm}H,\\ {\hat{C}}_{ij} & =\left[C_{2,ij}^{1}\;C_{3,ij}^{1}\;C_{4,ij}^{1}\right],\;\hat{A}=\left[A_{2,ij}^{1}\;A_{3,ij}^{1}\;A_{4,ij}^{1}\right],\;R_{g}=diag\left\{R_{g1},R_{g2},R_{g3}\right\},\\ H & =\left[I\;0\right],\;{\bar{C}}_{i}=\left[C_{i}\;0\right]. \end{array}$$

**Proof.**  Choose a piecewise Lyapunov function as follows:

(29) $$\begin{array}{c} \begin{array}{c} V_{g}\left(t\right)=V_{g}^{1}\left(t\right)+V_{g}^{2}\left(t\right)+V_{g}^{3}\left(t\right), \end{array} \end{array}$$

with

$$\begin{array}{cc} V_{g}^{1}\left(t\right) & =\psi^{T}\left(t\right)P_{g}\psi \left(t\right),\\ V_{g}^{2}\left(t\right) & =\sum_{m=0}^{2}\int_{t-h_{m}}^{t}e^{-\alpha (t-s)}\psi^{T}\left(t\right)Q_{gm}^{H}\psi \left(t\right)ds,\\ V_{g}^{3}\left(t\right) & =\sum_{m=0}^{2}h_{m}\int_{t-h_{m}}^{t}\int_{\theta }^{t}e^{-\alpha (t-s)}{\dot{\psi }}^{T}\left(t\right)R_{gm}^{H}\dot{\psi }\left(t\right)dsd\theta . \end{array}$$

Differentiating $V_{g}\left(t\right)$ in (29) yields:

(30) $$\begin{array}{cc} {\dot{V}}_{g}^{1}\left(t\right) & =2\psi^{T}\left(t\right)P_{g}\dot{\psi }\left(t\right),\\ {\dot{V}}_{g}^{2}\left(t\right) & =-\alpha V_{g}^{2}\left(t\right)+\sum_{m=0}^{2}\left(1-e^{-\alpha h_{m}}\right)\psi^{T}\left(t-h_{m}\right)Q_{gm}^{H}\psi \left(t-h_{m}\right),\\ {\dot{V}}_{g}^{3}\left(t\right) & \leq -\alpha V_{g}^{3}\left(t\right)+\sum_{m=0}^{2}h_{m}^{2}{\dot{\psi }}^{T}\left(t\right)R_{gm}^{H}\dot{\psi }\left(t\right)-\sum_{m=0}^{2}h_{m}\int_{t-h_{m}}^{t}{\dot{\psi }}^{T}\left(t\right)R_{gm}^{H}\dot{\psi (t)}ds. \end{array}$$

To analyze the $H_{-}$ fault sensitivity condition β and $H_{\infty }$ performance level γ, define $J_{1}\left(t\right)$ and $J_{2}\left(t\right)$ as:

(31) $$\begin{array}{cc} \; & J_{1}\left(t\right)=\beta^{2}f^{T}\left(t\right)f\left(t\right)-r_{e}^{T}\left(t\right)r_{e}\left(t\right), \end{array}$$

(32) $$\begin{array}{cc} \; & J_{2}\left(t\right)=r_{e}^{T}\left(t\right)r_{e}\left(t\right)-\gamma^{2}\rho^{T}\left(t\right)\rho \left(t\right). \end{array}$$

Assume that the fault occurs in the middle-frequency domain, and using Lemma 2, it follows that

(33) $$\begin{array}{cc} U & =\int_{0}^{+\infty }\left[\left(\omega_{1}\psi \left(t\right)+j\dot{\psi }\left(t\right)\right){\left(\omega_{2}\psi \left(t\right)+j\dot{\psi }\left(t\right)\right)}^{*}\right]dt\\ \; & =\frac{1}{2\pi }\int_{-\infty }^{+\infty }\left[\left(\omega_{1}-\omega \right)\left(\omega_{2}-\omega \right)X\left(\omega \right)X^{T}\left(\omega \right)\right]d\omega , \end{array}$$

where X denotes the Fourier transform. According to (9), it is obvious that τU≤0 holds for all solutions of (16), which is equivalent to (21). Note that $Q_{f}^{H}\geq 0$; then, we have $tr(UQ_{f}^{H})\leq 0$. Similar to the trace operations in [29], one can obtain that

(34) $$\begin{array}{cc} \; & tr\{\frac{Q_{f}^{H}}{2}He\left[\left(\omega_{1}\psi \left(t\right)+j\dot{\psi }\left(t\right)\right){\left(\omega_{2}\psi \left(t\right)+j\dot{\psi }\left(t\right)\right)}^{*}\right]\}\\ \; & =\psi^{T}\left(t\right)\omega_{1}\omega_{2}Q_{f}^{H}\psi \left(t\right)+\psi^{T}\left(t\right)j\omega_{0}Q_{f}^{H}\dot{\psi }\left(t\right)-{\dot{\psi }}^{T}\left(t\right)j\omega_{0}Q_{f}^{H}\psi \left(t\right)+{\dot{\psi }}^{T}\left(t\right)Q_{f}^{H}\dot{\psi }\left(t\right)\leq 0, \end{array}$$

with $\omega_{0}=1/2\left(\omega_{1}+\omega_{2}\right)$.

For $t\in H_{k,n}\cap L_{1,n}$, define $\varpi_{1}\left(t\right)=\mathrm{col}\left\{\psi \left(t\right),\psi \left(t-\epsilon \right),\psi \left(t-\epsilon -\frac{T}{2}\right),\psi \left(t-\epsilon -T\right),\psi \left(t-h\right),\psi \left(t-h-\frac{T}{2}\right),\psi \left(t-h-T\right),\vartheta \left(t\right),e_{k,n}\left(t\right)\right\}$. Combining (12), (27), (30)–(34), and Lemma 1 yields that

(35) $$\begin{array}{c} {\dot{V}}_{1}\left(t\right)+\alpha V_{1}\left(t\right)+J_{r}\left(t\right)\leq \sum_{i=1}^{2}\sum_{j=1}^{2}\varpi_{1}^{T}\left(t\right){\hat{\Phi }}_{ij}^{1r}\varpi_{1}\left(t\right), \end{array}$$

with ${\hat{\Phi }}_{ij}^{1r}=\Phi_{ij}^{1r}+\Delta_{ij}^{1}R_{1}^{-1}\Delta_{ij}^{1^{T}}+\Xi_{ij}^{1^{T}}\Xi_{ij}^{1}$.

Note that the membership functions satisfies

(36) $$\begin{array}{c} \sum_{i=1}^{2}\sum_{j=1}^{2}\varphi_{i}\left({\hat{\varphi }}_{j}-\varphi_{j}\right)\varpi_{1}^{T}\left(t\right){\hat{\Phi }}_{ij}^{1r}\varpi_{1}\left(t\right)=0. \end{array}$$

Combining (36) and ${\hat{\varphi }}_{j}\geq k_{j}\varphi_{j}$, it follows that

(37) $$\begin{array}{cc} \; & \sum_{i=1}^{2}\sum_{j=1}^{2}\varphi_{i}{\hat{\varphi }}_{j}\varpi_{1}^{T}\left(t\right){\hat{\Phi }}_{ij}^{1r}\varpi_{1}\left(t\right)\leq \sum_{i=1}^{2}\sum_{j=1}^{2}\varphi_{i}^{2}\varpi_{1}^{T}\left(t\right)\left[k_{i}\left({\hat{\Phi }}_{ij}^{1r}-M_{i}^{1r}\right)+M_{i}^{1r}\right]\varpi_{1}\left(t\right)\\ \; & +\sum_{i=1}^{2}\sum_{i<j}\varphi_{i}\varphi_{j}\varpi_{1}^{T}\left(t\right)\left[k_{i}\left({\hat{\Phi }}_{ij}^{1r}-M_{i}^{1r}\right)+M_{i}^{1r}+k_{j}\left({\hat{\Phi }}_{ji}^{1r}-M_{j}^{1r}\right)+M_{j}^{1r}\right]\varpi_{1}\left(t\right). \end{array}$$

Using the Schur complement to (26), it is easy to derive that ${\hat{\Phi }}_{ij}^{1r}<0$.

Likewise, for $t\in [\tau_{2,n},\tau_{1,n+1})$, defining $\varpi_{2}\left(t\right)=\mathrm{col}\left\{\psi \left(t\right),\psi \left(t-\epsilon \right),\psi \left(t-\epsilon -\frac{T}{2}\right),\psi \left(t-\epsilon -T\right),\psi \left(t-h\right),\psi \left(t-h-\frac{T}{2}\right),\psi \left(t-h-T\right),\vartheta \left(t\right)\right\}$, it holds that

(38) $$\begin{array}{c} {\dot{V}}_{2}\left(t\right)+\alpha V_{2}\left(t\right)+J_{r}\left(t\right)\leq \varpi_{2}^{T}\left(t\right){\hat{\Phi }}_{ij}^{2r}\varpi_{2}\left(t\right), \end{array}$$

with ${\hat{\Phi }}_{ij}^{2r}=\Phi_{ij}^{2r}+\Delta_{ij}^{2}R_{2}^{-1}\Delta_{ij}^{2^{T}}+\Xi_{ij}^{2^{T}}\Xi_{ij}^{2}$.

Let ϑ(t)≡0, and considering the arbitrary of *k*, one has

(39) $$\begin{array}{c} V_{g}\left(t\right)\leq e^{-\alpha (t-\tau_{g,n})}V_{g}\left(\tau_{g,n}\right),\;\forall t\in \left[\tau_{g,n},\tau_{3-g,n+g-1}\right). \end{array}$$

Combining (28) and (39), we can obtain

(40) $$\begin{array}{c} V_{g}\left(\tau_{g,n}\right)\leq \mu_{3-g}V_{3-g}\left(\tau_{g,n}^{-}\right). \end{array}$$

Based on the above discussion, adopting the similar recursive process in [37] yields

(41) $$\begin{array}{c} V\left(t\right)\leq \left\{\begin{array}{cc} V_{1}\left(0\right)e^{\delta -\frac{\delta }{T}t}, & t\in [\tau_{1,n},\tau_{2,n})\\ \frac{1}{\mu_{2}}e^{\alpha (T-T_{\mathrm{off}}^{\min})}e^{-\frac{\delta }{T}t}V_{1}\left(0\right), & t\in [\tau_{2,n},\tau_{1,n+1}), \end{array}\right. \end{array}$$

which implies

(42) $$\begin{array}{c} V\left(t\right)\leq \pi e^{-\frac{\delta }{T}t}V_{1}\left(0\right), \end{array}$$

with $\pi =\max\{e^{k},\left(1/\mu_{2}\right)e^{\alpha (T-T_{\mathrm{off}}^{\min})}\}$, $\delta =\alpha T-\mathrm{In}(\mu_{1}\mu_{2})>0$.

Recalling the definition of V(t), and denoting ${∥\begin{array}{c} \psi_{0} \end{array}∥}_{h}=\max\{∥\psi \left(t_{0}+s\right)∥,∥\dot{\psi }\left(t_{0}+s\right)∥\}$ for s∈[−h,0), then, it is easy to obtain that

(43) $$\begin{array}{c} V\left(t\right)\geq \xi_{1}{∥\begin{array}{c} \psi (t) \end{array}∥}^{2},\;V_{1}\left(0\right)\leq \xi_{2}{∥\begin{array}{c} \psi_{0} \end{array}∥}_{h}^{2}, \end{array}$$

with $\xi_{1}=\min\left\{\lambda_{\min}\left(P_{g}\right)\right\},\xi_{2}=\max\{\lambda_{\max}\left(P_{g}\right)+\sum_{m=0}^{2}\left\{\left(h_{m}^{3}/2\right)\lambda_{\max}\left(R_{gm}\right)+\lambda_{max}\left(Q_{gm}\right)\right\}$.

Combining (42) and (43) yields that

(44) $$\begin{array}{c} ∥\begin{array}{c} \psi (t) \end{array}∥\leq \sqrt{\frac{\pi \xi_{2}}{\xi_{1}}}e^{-\frac{\delta }{2T}t}{∥\begin{array}{c} \psi_{0} \end{array}∥}_{h}, \end{array}$$

which indicates that the system (16) is exponentially stable with the decay rate δ/2T.

In the following, the $H_{-}$ fault sensitivity and $H_{\infty }$ norm bound of the proposed system (16) will be proved, respectively.

For ρ(t)≡0 under zero initial conditions, it is obvious that $J_{1}\left(t\right)<0$ is equivalent to the $H_{-}$ condition in (20). Considering the fact that $V_{g}\left(+\infty \right)>0,V_{g}\left(0\right)=0$, integrating $J_{1}\left(t\right)$ from 0 to +∞, it follows that

(45) $$\begin{array}{cc} \; & \int_{0}^{\infty }J_{1}\left(t\right)dt=\int_{0}^{+\infty }\left[J_{1}\left(t\right)+{\dot{V}}_{g}\left(t\right)+\alpha V_{g}\left(t\right)\right]dt-V_{g}\left(+\infty \right)+V_{g}\left(0\right)-\int_{0}^{+\infty }\alpha V_{g}\left(t\right)dt\\ \; & \leq \int_{0}^{\infty }\left[J_{g}\left(t\right)+{\dot{V}}_{g}\left(t\right)+\alpha V_{g}\left(t\right)\right]dt\leq 0, \end{array}$$

which implies the system (16) satisfies the $H_{-}$ condition in (20).

For f(t)≡0 under zero initial conditions, employing a similar method, we have that if $J_{2}\left(t\right)<0$ holds, the $H_{\infty }$ performance objective in (22) is satisfied. By the same analysis as $J_{1}\left(t\right)$, one can obtain

(46) $$\begin{array}{cc} \; & \int_{0}^{\infty }J_{2}\left(t\right)dt\leq \int_{0}^{\infty }\left[J_{2}\left(t\right)+{\dot{V}}_{g}\left(t\right)+\alpha V_{g}\left(t\right)\right]dt\leq 0, \end{array}$$

which means that the system (16) has an $H_{\infty }$ norm bound γ in (22). This ends the proof. □

**Theorem** **2.** *For given positive constants T, $T_{\mathrm{off}}^{\min}$, $h<T_{\mathrm{off}}^{\min}$, ε, γ, α, β, $k_{i}$, and $\mu_{g}>1$, the system (16) is exponentially stable with an $H_{\infty }$ norm bound γ while also sensitive to the finite-frequency faults, if positive symmetric matrices $Q_{f}$, ${\tilde{P}}_{g}$, $Q_{gm}$, $R_{gm}$,* Ω*, W, and matrices ${\bar{A}}_{fj}$, ${\bar{B}}_{fj}$, ${\bar{C}}_{fj}$, ${\bar{D}}_{fj}$, $N_{i}^{gr}$, $G_{gm}$ exist with ${\tilde{P}}_{g}-W>0$, such that*

(47) $$\begin{array}{cc} \; & {\bar{\Pi }}_{ij}^{gr}<0,\;k_{i}{\bar{\Pi }}_{ij}^{gr}+k_{j}{\bar{\Pi }}_{ji}^{gr}+N_{i}^{gr}+N_{j}^{gr}<0\;\left(i<j\right), \end{array}$$

(48) $$\begin{array}{ll} \; & \left[\begin{array}{cc} R_{gm} & G_{gm}\\ {}* & R_{gm} \end{array}\right]>0, \end{array}$$

(49) $$\begin{array}{cc} \; & \left\{\begin{array}{c} {\tilde{P}}_{g}\leq \mu_{3-g}{\tilde{P}}_{3-g}\\ Q_{gm}\leq \mu_{3-g}Q_{(3-g)m}\\ R_{gm}\leq \mu_{3-g}R_{(3-g)m} \end{array}\right.,\;\alpha T-\mathrm{In}\left(\mu_{1}\mu_{2}\right)>0, \end{array}$$

*where*

Ψ¯ijgr=Φ¯ijgrΔ¯igΞ¯ijgT∗−Rg0∗∗−I,Π¯ijg1=Re(Ψ¯ijg1)Im(Ψ¯ijg1)−Im(Ψ¯ijg1)Re(Ψ¯ijg1)−Nig1,Π¯ijg2=Ψ¯ijg2−Nig2,Φ¯ij1r=Γ¯11i1rΓ¯12j1rΓ¯13ij1rΓ¯141rΓ¯15i1rB¯fj∗Γ¯22j1rΓ¯23ij1r0WB¯iB¯fj∗∗Γ¯33i1rΓ¯341r00∗∗∗Γ¯441r00∗∗∗∗Γ¯551r0∗∗∗∗∗−Ω,Φ¯ij2r=Γ¯11i2rΓ¯12j2rΓ¯13ij2rΓ¯142rΓ¯15i2r∗Γ¯22j2rΓ¯23ij2r0WB¯i∗∗Γ¯332rΓ¯342r0∗∗∗Γ¯442r0∗∗∗∗Γ¯552r,Λ¯i1=[A¯i000B¯i0],Λ¯i2=[A¯i000B¯i],Ξ¯ij1=[0C¯fjC^ij0H¯Dfj],Ξ¯ij2=[0C¯fj00H¯],Δ¯ig=[h0Λ¯igTRg0h1Λ¯igTRg1h2Λ¯igTRg2],Γ¯11ig1=Γ¯11ig2−w1w2Qf,Γ¯11ig2=αP˜g+[P˜gA¯i]s+∑i=02[Qgm−e−αhmRgm],Γ¯12jgr=αW+A¯fj,

$$\begin{array}{cc} {\bar{\Gamma }}_{13ij}^{gr} & =\left[{\bar{n}}_{0,ij}^{g}\;{\bar{n}}_{1,ij}^{g}\;{\bar{n}}_{2,ij}^{g}\right],\;{\bar{\Gamma }}_{14}^{gr}=\left[e^{-\alpha h_{0}}G_{g0}\;e^{-\alpha h_{1}}G_{g1}\;e^{-\alpha h_{2}}G_{g2}\right],\;{\bar{\Gamma }}_{15i}^{g2}={\tilde{P}}_{g}{\bar{B}}_{i},\\ {\bar{\Gamma }}_{15i}^{g1} & =\left[{\tilde{P}}_{g}-jw_{0}Q_{f}\right]{\bar{B}}_{i},\;{\bar{\Gamma }}_{22j}^{gr}=\alpha W+{\left[{\bar{A}}_{fj}\right]}_{s},\;{\bar{\Gamma }}_{23ij}^{gr}=\left[{\tilde{n}}_{0,ij}\;{\tilde{n}}_{1,ij}\;{\tilde{n}}_{2,ij}\right],\\ {\bar{\Gamma }}_{33i}^{1r} & =\left[\begin{array}{ccc} {\bar{J}}_{0,i}^{1} & \omega_{0,i} & \omega_{1,i}\\ {}* & {\bar{J}}_{1,i}^{1} & \omega_{2,i}\\ {}* & * & {\bar{J}}_{2,i}^{1} \end{array}\right],\;\omega_{m,i}=n_{m+1}^{2}\varepsilon C_{i}^{T}\Omega C_{i},\;{\bar{\Gamma }}_{34}^{gr}=diag\left\{{\bar{d}}_{g0},{\bar{d}}_{g1},{\bar{d}}_{g2}\right\},\\ {\bar{\Gamma }}_{33}^{2r} & =diag\left\{{\bar{J}}_{0}^{2},{\bar{J}}_{1}^{2},{\bar{J}}_{2}^{2}\right\},\;{\bar{\Gamma }}_{44}^{gr}=diag\left\{{\bar{r}}_{g}^{0},{\bar{r}}_{g}^{1},{\bar{r}}_{g}^{2}\right\},\;{\bar{\Gamma }}_{55}^{1r}=-\beta^{2}I,\;{\bar{\Gamma }}_{55}^{2r}=-\gamma^{2}I,\\ {\bar{n}}_{m,ij}^{g} & ={\tilde{n}}_{m,ij}+e^{-\alpha h_{m}}\left[R_{gm}-G_{gm}\right],\;{\tilde{n}}_{m,ij}=n_{m+1}{\bar{B}}_{fj}C_{i},\;{\bar{r}}_{g}^{m}=-e^{-\alpha h_{m}}\left[Q_{gm}+R_{gm}\right],\\ {\bar{J}}_{m,i}^{1} & =e^{-\alpha h_{m}}{\left[G_{1m}-R_{1m}\right]}_{s}+n_{m+1}^{2}\varepsilon \omega_{i},\;{\bar{J}}_{m}^{2}=e^{-\alpha h_{m}}{\left[G_{2m}-R_{2m}\right]}_{s}. \end{array}$$

*Moreover, the filter gains are given by*

(50) $$\begin{array}{cc} A_{fj} & ={\bar{A}}_{fj}W^{-1},\;B_{fj}={\bar{B}}_{fj},\;C_{fj}={\bar{C}}_{fj}W^{-1},\;D_{fj}={\bar{D}}_{fj}. \end{array}$$

**Proof.**  Suppose $P_{g}=\left[\begin{array}{cc} {\tilde{P}}_{g} & X\\ {}* & Z \end{array}\right]$, and define $F_{1}=\left\{I,XZ^{-1},I,I,I,I,I,I\right\}$, $F_{2}=\{I,XZ^{-1},$

I,I,I,I,I}. Pre- and post-multiplying $\Psi_{ij}^{gr}$ with $F_{g}$ and its transpose yields ${\bar{\Psi }}_{ij}^{gr}$, with new variables denoted as follows:

$$\begin{array}{cc} {\bar{A}}_{fj} & =XA_{fj}Z^{-1}X^{T},\;{\bar{B}}_{fj}=XB_{fj},\;W=XZ^{-1}X^{T}\\ {\bar{C}}_{fj} & =C_{fj}Z^{-1}X^{T},\;{\bar{D}}_{fj}=D_{fj}. \end{array}$$

Similarly to (36), it holds that

(51) $$\begin{array}{c} \sum_{i=1}^{2}\sum_{j=1}^{2}\varphi_{i}\left({\hat{\varphi }}_{j}-\varphi_{j}\right)\left({\bar{\Pi }}_{ij}^{gr}+N_{i}^{gr}\right)=0. \end{array}$$

Combining (47), (51), and ${\hat{\varphi }}_{j}\geq k_{j}\varphi_{j}$, it follows that

(52) $$\begin{array}{cc} \; & \sum_{i=1}^{2}\sum_{j=1}^{2}\varphi_{i}{\hat{\varphi }}_{j}\left({\bar{\Pi }}_{ij}^{gr}+N_{i}^{gr}\right)\leq \sum_{i=1}^{2}\sum_{j=1}^{2}\varphi_{i}^{2}\left[k_{i}{\bar{\Pi }}_{ij}^{gr}+N_{i}^{gr}\right]\\ \; & +\sum_{i=1}^{2}\sum_{i<j}\varphi_{i}\varphi_{j}\left[k_{i}{\bar{\Pi }}_{ij}^{gr}+N_{i}^{gr}+k_{j}{\bar{\Pi }}_{ij}^{gr}+N_{j}^{gr}\right]\leq 0. \end{array}$$

By Lemma 3 and (52), we have ${\bar{\Psi }}_{ij}^{gr}<0$, which is equivalent to $\Psi_{ij}^{gr}<0$. Then, it holds that ${\hat{\Phi }}_{ij}^{gr}<0$ by the Schur complement. In addition, $P_{g}>0$ is equivalent to ${\tilde{P}}_{g}-XZ^{-1}X^{T}>0$, i.e., ${\tilde{P}}_{g}-W>0$. Therefore, by following the similar proof process in Theorem 1, the exponential stability of the system (16) can be obtained, along with the $H_{\infty }$ attenuation level γ and the $H_{-}$ sensitivity condition β.

Using the similar approach in [32], the FDF gains can be calculated as in (50). This ends the proof. □

## 4. Simulation

A DC microgrid with one CPL is presented in this section, where the circuit parameters are set as: $r_{L,1}=0.8\Omega$, $r_{s}=0.4\Omega$, $L_{1}=40$ mH, $C_{1}=1$ mf, $L_{s}=17.3$ mH, $C_{s}=1.05$ mf, $P_{1}=450$ W, $V_{d}c=200$ V. Choose the reference voltage $v_{c0,1}$ as 300 V, and ${\tilde{w}}_{2,1}$ as 100 V. The fuzzy membership functions are given by:

(53) $$\begin{array}{c} \left\{\begin{array}{c} \varphi_{1}\left(t\right)=2-\frac{400}{300+{\tilde{v}}_{C,1}\left(t\right)}\\ \varphi_{2}\left(t\right)=1-\varphi_{1}\left(t\right) \end{array}\right.. \end{array}$$

Choose the system controller ${\tilde{K}}_{i}$ as ${\tilde{K}}_{1}={\tilde{K}}_{2}=[-0.0389$ −0.0002 0.0016 −0.0007]. The initial states of the DC microgrids and the FDF are given by $x^{T}\left(0\right)=[-0.003$ 0.283 0.28 0.28], and ${\hat{x}}^{T}\left(0\right)=[0$ 0 0 0], respectively.

The external disturbance ρ(t) and the finite-frequency fault f(t) are given by:

(54) $$\begin{array}{cc} \rho (t)= & \left\{\begin{array}{cc} 10e^{-7.5t}sin\left(50t\right),\; & t\in [0,1]\\ 0,\; & \mathrm{otherwise}, \end{array}\right., \end{array}$$

(55) $$\begin{array}{cc} f(t)= & \left\{\begin{array}{cc} 10e^{-0.1t}sin\left(8\pi t\right),\; & t\in [0.2,0.5]\\ 0,\; & \mathrm{otherwise}. \end{array}\right. \end{array}$$

Set α=0.4, $\mu_{1}=\mu_{2}=1.02$, T=0.1 s, $T_{\mathrm{off}}^{\min}=0.025$ s, h=0.001 s, ε=0.05, $k_{i}=k_{j}=0.5$, $\omega_{1}=3$ $s^{-1}$, $\omega_{2}=5$ $s^{-1}$, T=0.001 s, γ=10, β=1.225. By solving Theorem 2, the gain matrices of the proposed FDF and the event-triggered matrix Ω are obtained:

$$\begin{array}{cc} A_{f_{1}} & =10^{-4}\times \left[\begin{array}{cccc} 0.0001 & -0.1445 & -0.0001 & 0.0004\\ 0.0012 & -0.1434 & 0.0000 & -0.0005\\ -0.0001 & -0.0021 & 0.0001 & -0.0007\\ -0.0006 & -0.0021 & 0.0003 & -0.0009 \end{array}\right],\\ A_{f_{2}} & =10^{-4}\times \left[\begin{array}{cccc} 0.0001 & -0.1290 & -0.0001 & 0.0003\\ 0.0008 & -0.1298 & 0.0000 & -0.0004\\ -0.0001 & -0.0019 & 0.0001 & -0.0006\\ -0.0005 & -0.0019 & 0.0003 & -0.0008 \end{array}\right],\\ B_{f_{1}} & ={\left[\begin{array}{cccc} 0.0065 & -0.0012 & 0.0038 & 0.0007 \end{array}\right]}^{T},\\ B_{f_{2}} & ={\left[\begin{array}{cccc} 0.0084 & -0.0015 & 0.0049 & 0.0009 \end{array}\right]}^{T},\\ C_{f_{1}} & =10^{-4}\times \left[\begin{array}{cccc} -0.0401 & -0.3097 & -0.0399 & -0.0477 \end{array}\right],\\ C_{f_{2}} & =10^{-4}\times \left[\begin{array}{cccc} -0.0242 & -0.1873 & -0.0242 & -0.0288 \end{array}\right],\\ D_{f1} & =-0.0062,\;D_{f2}=-0.0231,\;\Omega =0.0027. \end{array}$$

**Figure 2:**
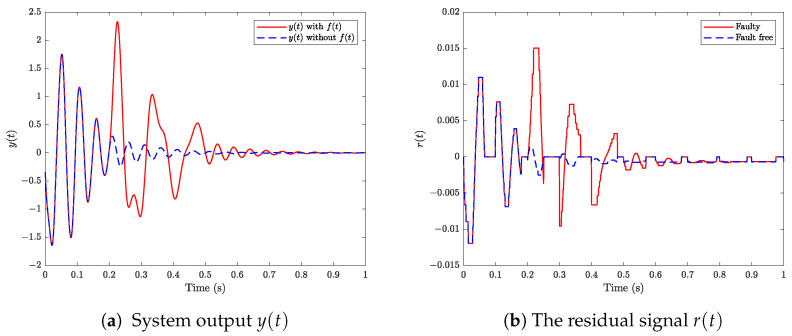
Impacts of f(t) on the system output y(t) and the residual signal r(t).

Figure 2 illustrates the output behavior of the DC microgrid under two conditions: without and with the fault f(t) described in (55). The comparison reveals a significant impact of the fault on the system dynamics. Utilizing such output for control feedback poses challenges in achieving system stability. Hence, prompt detection of this fault is imperative to prevent damage from the DC microgrid system. Moreover, note that the generated signal r(t) changes greatly when the fault occurs at t=0.2s, which is helpful for the fault detection.

**Figure 3:**
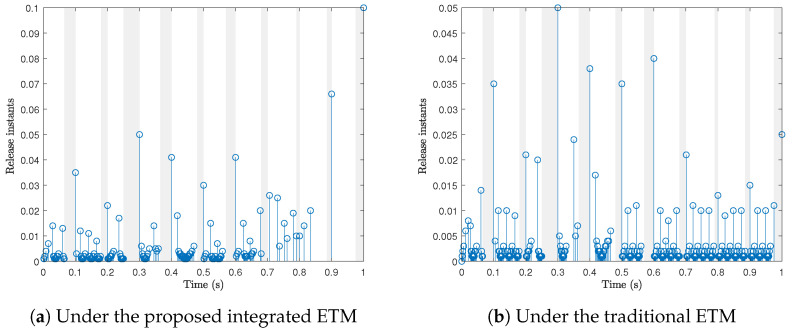
Release instants under different triggering mechanisms.

Figure 3 displays the release instants and releasing intervals of the system under the proposed integrated ETM and the traditional ETM, respectively. Table 1 records the data releasing rate under the above two ETMs. It can be observed that the data releasing rate under our proposed integrated ETM is 14.4%, which is much less than the one under the traditional ETM, thus saving the limited network resource.

**Figure 4:**
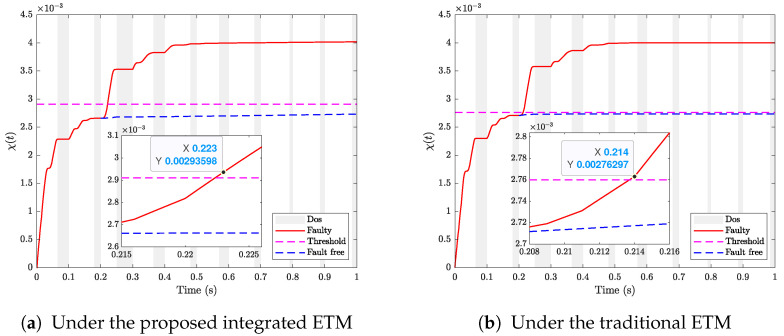
Fault detection performance under different triggering mechanisms.

Figure 4 depicts the fault detection performance with different triggering mechanisms, from which we can observe that by the proposed ETM, the fault is detected at t=0.223 s, which is slower than the traditional ETM (t=0.214 s), indicating that although our integrated ETM can further reduce the resource utilization of the network, there still exists a trade-off between the fault detection performance and the amount of releasing data.

**Figure 5:**
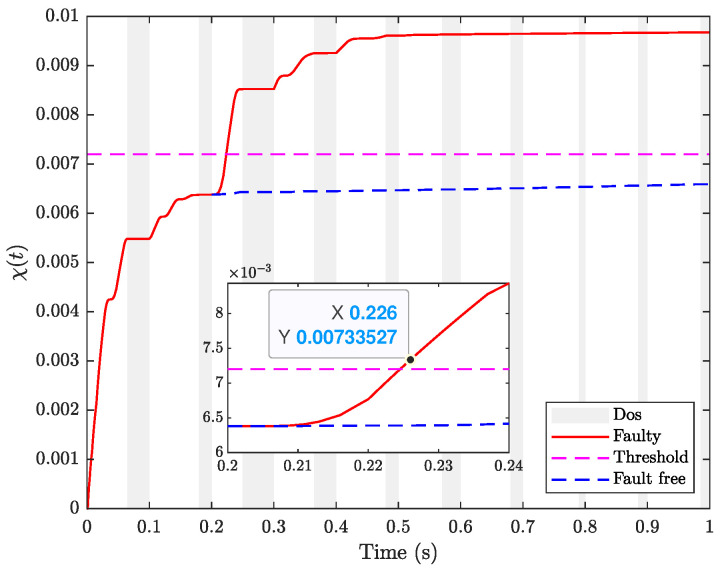
Fault detection performance using $H_{\infty }$ method.

From Figure 5, it is observed that the general $H_{\infty }$ FDF detects the fault until t=0.226 s, which is slower than the proposed method for the fault detection in Figure 4 (t=0.223 s). The proposed FDF method in this study is designed for the fault occurring within a specified frequency band, thereby exhibiting less conservatism compared to general $H_{\infty }$ methods designed to detect faults across the entire frequency.

## 5. Conclusions

A resilient event-based $H_{-}/H_{\infty }$ FDF for DC microgrids against DoS attacks has been designed in this paper, where the actuator fault is assumed to occur in a finite-frequency domain, reducing more conservatism in the FDF design. The T-S fuzzy model is used to handle nonlinear CPLs in DC microgrids, and a novel integrated ETM is proposed to further decrease the unnecessary triggering events compared to the traditional ETM. By constructing the switched residual system based on fuzzy rules, sufficient conditions of exponential stability are obtained, along with the $H_{\infty }$ attenuation bound and $H_{-}$ sensitive condition. The simulation results demonstrate the FDF’s ability to rapidly detect finite-frequency faults, even in the presence of a DoS attack. Notably, its superior performance compared to the conventional $H_{\infty }$ method underscores the effectiveness of the proposed approach. For future research, fault detection against hybrid attacks for DC microgrids requires deeper investigation.

References yes

## Figures and Tables

**Table 1:** The DRR under differents ETMs.

Cases	Traditional ETM	Our Integrated ETM
Total sampling	1000	1000
Total releasing	297	144
DRR	29.7%	14.4%

## Data Availability

The data presented in this study are available on request from the corresponding author.
